# Implementing nudges to promote utilization of low tidal volume ventilation (INPUT): a stepped-wedge, hybrid type III trial of strategies to improve evidence-based mechanical ventilation management

**DOI:** 10.1186/s13012-021-01147-7

**Published:** 2021-08-10

**Authors:** Meeta Prasad Kerlin, Dylan Small, Barry D. Fuchs, Mark E. Mikkelsen, Wei Wang, Teresa Tran, Stefania Scott, Aerielle Belk, Jasmine A. Silvestri, Tamar Klaiman, Scott D. Halpern, Rinad S. Beidas

**Affiliations:** 1 Pulmonary, Critical Care and Allergy Division, Perelman School of Medicine, University of Pennsylvania, Philadelphia, PA USA; 2 Department of Medicine, Perelman School of Medicine, University of Pennsylvania, Philadelphia, PA USA; 3 Palliative and Advanced Illness Research (PAIR) Center, Perelman School of Medicine, University of Pennsylvania, Philadelphia, PA USA; 4 Leonard Davis Institute of Health Economics, University of Pennsylvania, Philadelphia, PA USA; 5 Department of Statistics, The Wharton School, University of Pennsylvania, Philadelphia, PA USA; 6 Center for Health Incentives and Behavioral Economics (CHIBE), University of Pennsylvania, Philadelphia, PA USA; 7 Department of Medical Ethics and Health Policy, Perelman School of Medicine, University of Pennsylvania, Philadelphia, PA USA; 8 Department of Psychiatry, Perelman School of Medicine, University of Pennsylvania, Philadelphia, PA USA; 9 Penn Implementation Science Center at the Leonard Davis Institute of Health Economics (PISCE@LDI), University of Pennsylvania, Philadelphia, PA USA

**Keywords:** Hybrid implementation-effectiveness trial, Behavioral economics, Nudge, Mechanical ventilation, Acute respiratory distress syndrome, Low tidal volume

## Abstract

**Background:**

Behavioral economic insights have yielded strategies to overcome implementation barriers. For example, default strategies and accountable justification strategies have improved adherence to best practices in clinical settings. Embedding such strategies in the electronic health record (EHR) holds promise for simple and scalable approaches to facilitating implementation. A proven-effective but under-utilized treatment for patients who undergo mechanical ventilation involves prescribing low tidal volumes, which protects the lungs from injury. We will evaluate EHR-based implementation strategies grounded in behavioral economic theory to improve evidence-based management of mechanical ventilation.

**Methods:**

The Implementing Nudges to Promote Utilization of low Tidal volume ventilation (INPUT) study is a pragmatic, stepped-wedge, hybrid type III effectiveness implementation trial of three strategies to improve adherence to low tidal volume ventilation. The strategies target clinicians who enter electronic orders and respiratory therapists who manage the mechanical ventilator, two key stakeholder groups. INPUT has five study arms: usual care, a default strategy within the mechanical ventilation order, an accountable justification strategy within the mechanical ventilation order, and each of the order strategies combined with an accountable justification strategy within flowsheet documentation. We will create six matched pairs of twelve intensive care units (ICUs) in five hospitals in one large health system to balance patient volume and baseline adherence to low tidal volume ventilation. We will randomly assign ICUs within each matched pair to one of the order panels, and each pair to one of six wedges, which will determine date of adoption of the order panel strategy. All ICUs will adopt the flowsheet documentation strategy 6 months afterwards. The primary outcome will be fidelity to low tidal volume ventilation. The secondary effectiveness outcomes will include in-hospital mortality, duration of mechanical ventilation, ICU and hospital length of stay, and occurrence of potential adverse events.

**Discussion:**

This stepped-wedge, hybrid type III trial will provide evidence regarding the role of EHR-based behavioral economic strategies to improve adherence to evidence-based practices among patients who undergo mechanical ventilation in ICUs, thereby advancing the field of implementation science, as well as testing the effectiveness of low tidal volume ventilation among broad patient populations.

**Trial registration:**

ClinicalTrials.gov, NCT04663802. Registered 11 December 2020.

Contributions to the literature

- This protocol describes a stepped-wedge trial of simple and scalable behavioral economic strategies embedded within the electronic health record to improve adherence to evidence-based practices for lung protective ventilation.
- This trial will provide comparative evidence for default and accountable justification strategies.
- This study will generate evidence regarding the effectiveness and safety of low tidal volume ventilation among broad populations of patients who undergo mechanical ventilation.

## Introduction

Behavioral economics research—an approach to understanding decision-making and behavior that integrates behavioral science with economic principles—has yielded several promising strategies to overcome common barriers to implementation of evidence-based practices. As such, the intersection between behavioral economics and implementation research holds great promise as a rich new area of inquiry that has the potential to advance the science of implementation [1]. “Nudges” are behavioral economic strategies that change the decision-making environment to predictably influence choice, but without restricting it. Nudges trade off effectiveness with autonomy, such that the least intrusive strategies (for example, providing information to guide a choice) are least likely to result in promoting a particular decision or behavior [2]. Two examples of nudges near the top of the intervention ladder—that is, most effective but also most intrusive—include the “default” and “accountable justification” strategies. The first strategy is one that sets the desirable practice as the default unless a chooser actively opts out. Therefore, a default strategy harnesses the tendency for humans to proceed with the “status quo” and reduces friction and cognitive load to make the easiest decision. It can also indirectly overcome lack of knowledge—a commonly identified barrier—about those desirable practices by providing an easy path to accurately employing those practices [3, 4]. In the second strategy, choosers must provide an explicit rationale if they deviate from a desirable practice (i.e., provide accountable justification). This signals an injunctive norm—what “should” be done—and thereby exerts social pressure that creates an implicit default. Although the injunctive norm function of requiring justification for undesirable behaviors is generally a weaker nudge than a true default, the approach can be strengthened considerably by also incorporating social accountability through making the justifications part of a record that can be used for auditing, feedback, and judgment by peers [5–8]. Accountable justification also preserves a greater degree of discretion and is therefore less intrusive than defaults, potentially increasing acceptability when a treatment is not universally appropriate for a heterogeneous population, and better enabling clinical judgment to influence outcomes.

Embedding default and accountable justification strategies in the electronic health record (EHR) can be a simple and scalable way to create implementation strategies that can be scaled up for dissemination. Furthermore, they are perceived to be feasible and acceptable [9]. Successful examples include strategies to increase ordering of generic rather than branded medications [10] and to reduce ordering of unnecessary computerized tomography (CT) scans [11] and inappropriate antibiotic prescribing [12]. Therefore, the incorporation of behavioral economic strategies in the EHR holds great promise for advancing the science of implementation.

We sought to apply these principles to the care of patients who undergo invasive mechanical ventilation (MV), a life-saving but also potentially injurious intervention administered to up to a million Americans annually within intensive care units (ICUs) [13–16]. Lung-protective ventilation (LPV) aims to minimize harm by delivering low tidal volumes (the amount of air per breath delivered by the ventilator) and limiting the artificial pressures in the lungs. LPV was first proven effective in patients with acute respiratory distress syndrome (ARDS), a severe form of respiratory failure associated with pneumonia, sepsis, and other common illnesses [17]. In 2001, a multicenter randomized trial demonstrated an absolute mortality reduction of 10%, shortened duration of MV, and reduced organ failure rates [18]. These findings have since been replicated and incorporated into international guidelines [19]; however, 15 years later, more than one-third of ARDS patients overall, and up to 81% in some ICUs, do not receive LPV [20, 21]

Multiple studies across diverse ICUs have consistently identified two key barriers to LPV utilization: knowledge gaps in ICU clinicians about administration of LPV [22–26] and tendencies to prescribe LPV only when ARDS is definitively diagnosed out of concern that LPV will require higher doses and longer duration of sedative medications to counteract the discomfort of non-physiologic ventilation [23, 24]. However, definitive diagnosis of ARDS is challenging [27, 28], and in fact, a growing body of evidence suggests that LPV reduces lung injury and mortality even among some patients without ARDS [29, 30]. In a recent randomized trial among a heterogeneous group of patients without ARDS, there was no difference in mortality or duration of MV; however, there was also no difference in the purported adverse effects of LPV, including sedative administration or days with delirium [31]. Taking together, the high-quality evidence of potential benefits of LPV among many patients who may be difficult to identify, lack of harm among broad patient populations, and the possibility of inertia in changing tidal volumes after establishing the initial settings [32], a preferred approach to implementation may be to favor LPV at the start among most or all patients who undergo MV. Applying behavioral economic strategies to the process of ordering and documenting MV in the electronic health record may support this approach.

The overall objective of the Implementation of Nudges to Promote Utilization of low Tidal volume ventilation (INPUT) study is to evaluate three electronic health record (EHR)-based implementation strategies grounded in behavioral economic theory to increase utilization of LPV in mechanically ventilated patients. This study will use a hybrid type III effectiveness implementation trial design to evaluate both implementation (primary) and effectiveness (secondary) outcomes [33]. As such, it will advance the science of both implementation in critical care and the application of behavioral economics to clinician decision-making, and it will also expand the evidence for effectiveness of LPV among diverse patient populations.

## Methods

### Study aims and implementation frameworks

**Fig. 1:**
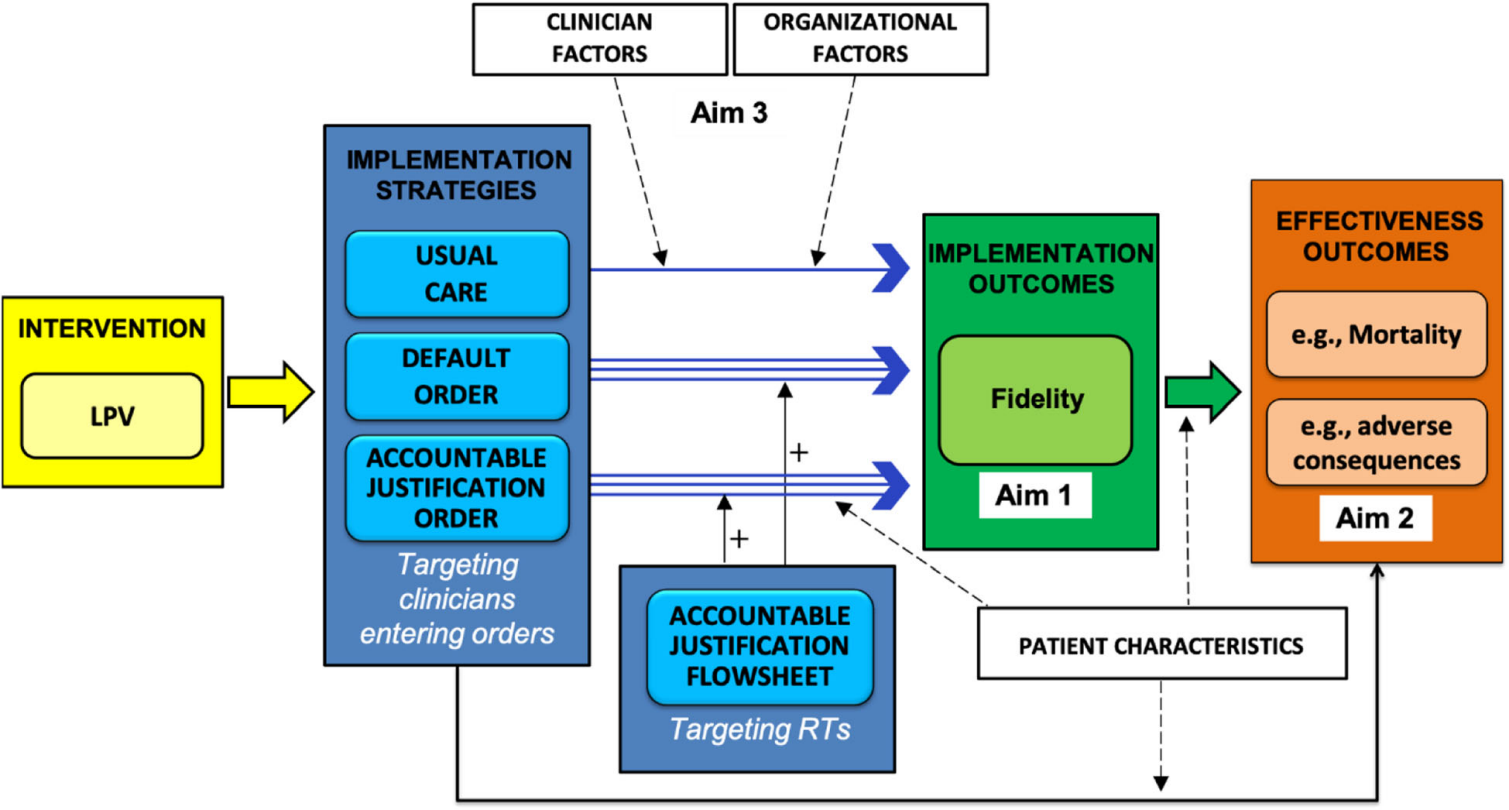
Conceptual model of relationships under study. The conceptual model maps the relationships under study to the Proctor model for implementation research. Dashed arrows indicated factors that may interact with the implementation strategies with respect to implementation and effectiveness outcomes. Aim 1 will test implementation outcomes, and aim 2 will test effectiveness outcomes

Figure 1 illustrates the conceptual model of the relationships between the implementation strategies under study in this trial and the implementation and effectiveness outcomes, based on the Proctor model for implementation research and the Consolidated Framework for Implementation Research (CFIR), both of which guided our study design [34, 35]. The first aim of the trial is to compare the effectiveness of the implementation strategies on the implementation outcome of fidelity to the evidence-based intervention of LPV. Fidelity to LPV will be defined as the percentage of time during the first 72 h of MV that a patient had tidal volumes documented as 6.5 ml/kg of their predicted body weight (ml/kg PBW), based on the primary randomized trial that demonstrated the effectiveness of LPV [18] and subsequent evidence of the association of early administration of low tidal volumes with better patient outcomes [32]. The second aim is to compare the effectiveness of the implementation strategies with respect to clinical outcomes, including in-hospital mortality, discharge disposition, duration of MV, duration of ICU and hospital stay, and potential adverse consequences associated with LPV. The third aim of the study is to assess how the strategies interact with clinician and environmental contextual elements, guided by the CFIR, such as the individual’s knowledge of LPV and experience caring for MV patients and the culture of collaboration between ordering clinicians and respiratory therapists (RTs) [35].

### Overview of study design

**Fig. 2:**
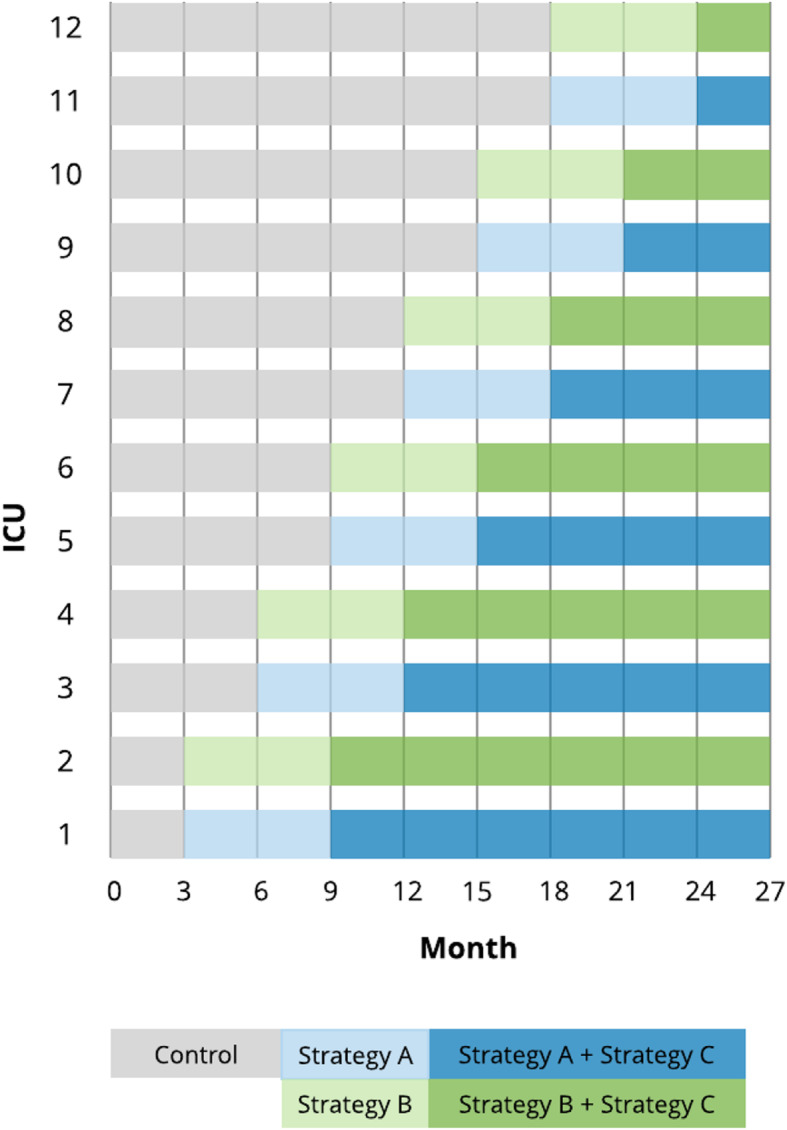
Trial schematic. Stepped wedge roll-out of three strategies to promote the use of lung-protective ventilation. Strategy A is a default order panel for MV that is auto-populated with lung-protective settings. Strategy B is an order panel that incorporates accountable justification requiring a user to provide a reason when LPV settings are not entered. Strategy C is an accountable justification strategy embedded in the flowsheet documentation

We will conduct a 5-arm, pragmatic, stepped-wedge, cluster randomized trial across 12 academic and community ICUs. Currently and prior to the trial, study ICUs variably use an EHR-based algorithm to identify patients with ARDS and prompt physicians to employ LPV via a daily data dashboard, which will serve as the control arm. During the study, ICUs will each add either a default order panel (strategy A) or an accountable justification order panel (strategy B), both of which target clinicians who enter orders for MV. Six months after deployment of the assigned order strategy, an accountable justification strategy will be added to the flowsheet documentation, which targets RTs (strategy C). Thus, the five study arms will be usual care, strategy A alone, strategy B alone, strategies A + C, and strategies B + C. Because of variability in study ICUs, we created pairs matched on patient volume and baseline rate of adherence to LPV to improve balance among study groups. Within matched pairs, each ICU will be assigned to strategy A or B, and matched pairs will be assigned to one of six wedges, which will determine the dates on which they initiate the order strategies. Both random assignments steps will use computerized random-number generation. The first wedge will begin at the start of the fourth month of the trial phase, so that all hospitals will contribute a minimum of 3 months of data prior to having adopted the implementation strategy. By the completion of the 27-month trial, all ICUs with contribute a minimum of 3 months of data with both assigned strategies in place. Figure 2 provides a schematic overview for the trial design.

### Study setting and populations

The study will take place in 12 ICUs nested with 5 hospitals of the University of Pennsylvania Health System. Hospitals are geographically and organizationally separate, and study ICUs have independent medical directors, diverse study populations, and variable bedside staffing models. (Table 1). All patients ages 18 and older admitted to a participating ICU during the study period who undergo MV via a mandatory mode for any period of time will be eligible for inclusion. We will exclude patients who meet any of the criteria listed in Table 2. All clinicians who interact with the implementation strategies in study ICUs and ICU directors will be eligible for participating in semi-structured interviews and electronic surveys, as detailed below.

**Table 1:** Characteristics of study ICUs

Hospital	ICU	Number of beds	ICU type	Ordering clinicians
A	1	24	General	Intensivists, APPs
B	2	24	General	Intensivists, APPs
C	3	29	General	APPs, residents
D	4	16	Medical	Hospitalists, APPs
	5	20	Surgical	APPs, residents
	6	20	Cardiac	APPs, residents
	7	20	Neurological	APPs, residents
E	8	24	Medical	Residents
	9	8	Medical	APPs
	10	24	Surgical	APPs, residents
	11	32	Cardiac surgical	APPs, residents
	12	22	Neurological	APPs, residents

*Abbreviations*: *APPs* Advanced practice providers

**Table 2:** Exclusion criteria

Criterion	Rationale for exclusion
The episode of MV lasts less than 12 h.	The evidence supporting low tidal volume ventilation does not apply to patients who undergo very short periods of MV, nor does it alter their outcomes.
The patient is on minimal settings for the entirety of MV, defined as a spontaneous mode (e.g., pressure support ventilation) with pressure support < 10 cmH_2_O, and PEEP < 8 cmH_2_0, and FiO_2_ < 50%.	The clinical significance of spontaneous tidal volumes is unknown and low tidal volumes may not be beneficial or desirable.
Goals of care are documented as comfort measures only during the first 72 h during episode of MV.	MV is managed differently during care focused exclusively on comfort and low tidal volume ventilation may not be appropriate, nor would it be expected to influence clinical outcomes.
There is no height documented in the EHR at the time of initiation of MV.	We will be unable to estimate ideal body weight, a necessary parameter to calculate the primary outcome, and patient’s without a documented height will not receive the interventions.

*Abbreviations: MV* Mechanical ventilation, *PEEP* Positive end expiratory pressure, *FiO*_*2*_ Fractional inspired oxygen

### Implementation strategies

**Fig. 3:**
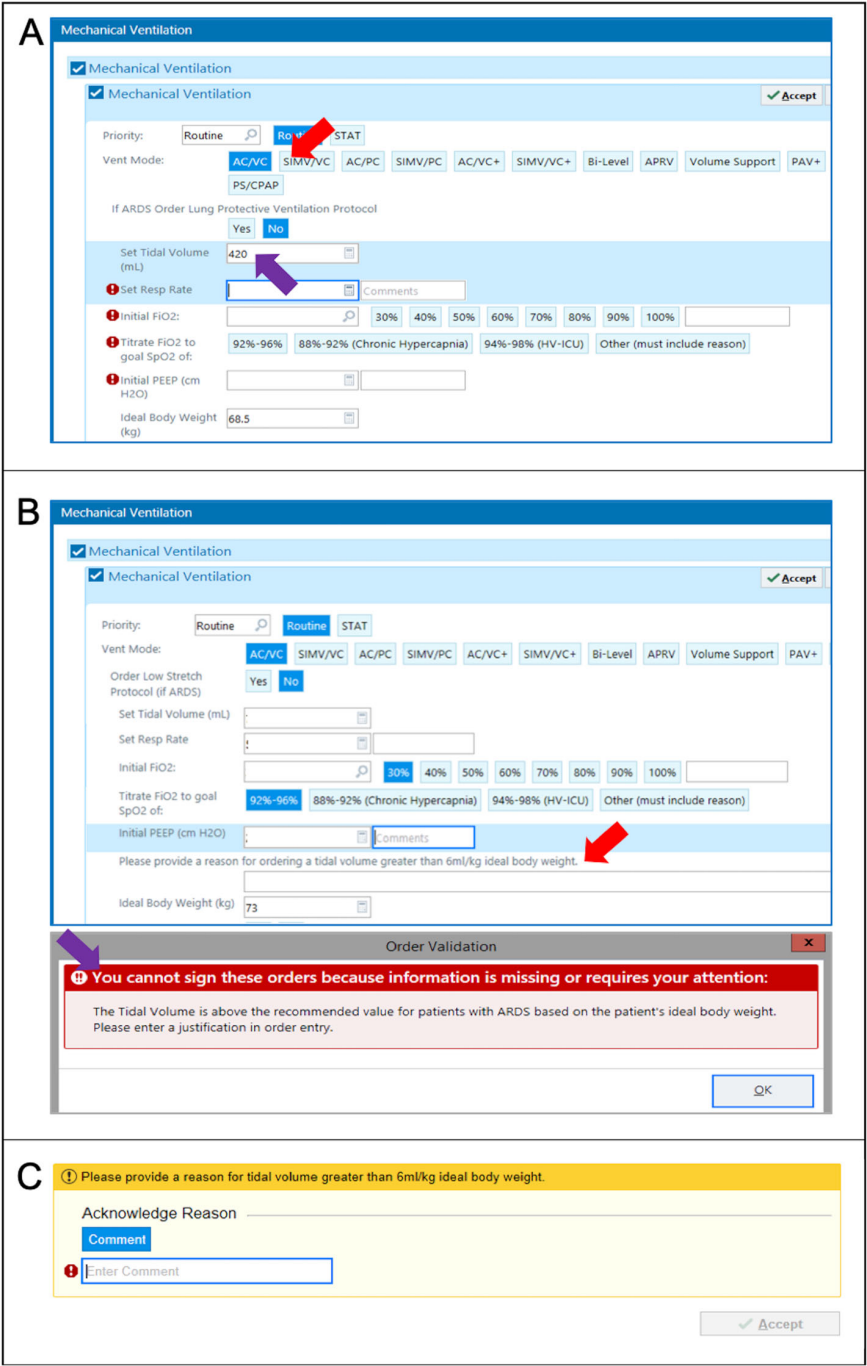
Screenshots of EHR-based strategies. **A** The default order strategy, in which the ventilation mode (red arrow) and tidal volume (purple arrow) are pre-populated. **B** The accountable justification order strategy, in which a reason must be provided (red arrow) if the set tidal volume entered is above 6.5 ml/kg PBW. If the reason is not provided upon order submission, an alert pops up and prevents order submission until the field is completed. **C** The accountable justification flowsheet strategy, in which a reason must be provided by the RT if a set tidal volume is documented to be above 6.5 ml/kg PBW

The trial will test three implementation strategies to promote the use of low tidal volume ventilation based on behavioral economics and built into the EHR. Strategy A (Fig. 3), the default order panel, targets ordering clinicians (physicians, physician assistants, and nurse practitioners) by pre-selecting the ventilation mode as assist-control, volume-cycled, and pre-populating the tidal volume via an automatic calculation of 6 ml/kg PBW, as determined by each patient’s height and gender. The ordering clinician can opt out of any of the pre-specified settings with one click.

Strategy B, the accountable justification order panel, also targets ordering clinicians. It has no pre-specified settings; however, if an ordering clinician enters a set tidal volume that is greater than 6.5 ml/kg PBW, they will be required to enter a reason into a free text box. The clinician will not be able to proceed with entering the MV order until after a response is entered and will be reminded by a message within the order panel that their response will be saved.

Strategy C uses accountable justification within the flowsheet documentation for MV, thereby targeting RTs. If an RT enters a set tidal volume value greater than 6.5 ml/kg into the flowsheet, they will receive a prompt to enter a reason into a free text box, similar to the physician-targeted accountable justification strategy. They will not be able to proceed with further documentation until after a response is entered. The prompt will fire once per patient per RT. Thus, if a patient is cared for by three RTs, for example, there may be as many as three separate prompts, depending on whether each RT documents a set tidal volume above the threshold. All flowsheet documentation that occurs while a patient is located within a study ICU will be exposed to strategy C.

### Study outcomes

We will employ a hybrid type III design to study both implementation (primary) and effectiveness (secondary) outcomes [36] We chose this design because of the very strong evidence base for the effectiveness of LPV in ARDS patients, and safety in patients without ARDS, plus evidence of a clear gap between these data and practice. Thus, there is both a strong need for implementation research and a need for further effectiveness evidence, particularly among all patients undergoing MV when ARDS criteria need not be met for the intervention to apply.

The primary study outcome will be fidelity to LPV, measured as the percentage of time during the first 72 h of MV during which a patient has tidal volumes of less than or equal to 6.5 ml/kg PBW. We chose this threshold because of the original randomized trial that demonstrated that targeting tidal volumes of 6 ml/kg PBW reduced mortality compared to higher tidal volumes; because the average tidal volumes administered in that trial in the study group was 6.4 ml/kg PBW; and to allow a small margin for rounding off the estimates of desirable tidal volume. We will limit the estimation to the first 72 h of MV due to existing evidence that the early course of MV is most impactful on clinical outcomes [32, 37]. We will also include the following secondary measures of adherence to LPV: initial tidal volume administered; duration of time exposed to plateau pressure (Pplat) > 30 mmHg; and durations of exposure to tidal volume > 8 ml/kg PBW (the upper limit of recommended tidal volumes for LPV [19]).

Table 3 details the effectiveness outcomes, including in-hospital mortality, hospital discharge disposition, duration of MV, ICU and hospital length of stay (LOS). We will also evaluate potential adverse consequences, including incidence of life-threatening acidemia, total cumulative doses of sedative medications during and after MV, and total number of days with acute brain dysfunction [38] during and after MV.

**Table 3:** Effectiveness outcomes and measures of adverse consequences

Outcome category	Outcome measure	Definition
Effectiveness	In-hospital mortality	Vital status at hospital discharge
	Discharge disposition	Binary variable for whether or not the patient was discharged to home
	Duration of MV	Total number of hours from trial enrollment to extubation for at least 24 h
	ICU length of stay	Total number of days from trial enrollment to discharge from study ICU
	Hospital length of stay	Total number of days from trial enrollment to discharge from hospital
Adverse consequences	Life-threatening acidemia	Arterial or venous blood gas during MV with a pH of less than 7.10
	Cumulative doses of sedative medications	Total doses of benzodiazepines, narcotics, or Propofol administered during MV as continuous infusions or bolus doses, calculated using dose equivalents in each class
	Days with acute brain dysfunction	Total number of days from the time of enrollment until hospital discharge with at least one of the following: a positive delirium screen using the Confusion Assessment Method for the ICU (CAM-ICU) or a Richmond Agitation-Sedation Scale (RASS) score less than or equal to − 4

### Assessment of contextual elements

To explore how the strategies interact with clinician and environmental contextual elements, we will conduct voluntary, semi-structured interviews with ICU clinicians (ordering clinicians and RTs) during the first 3 months after strategy implementation in each study ICU. A research coordinator trained in qualitative interviewing will use a semi-structured script guided by several constructs of the CFIR domains of characteristics of the individual, inner setting, and process [35] to elicit perceptions of the implementation strategies and identify factors that may influence the success of the strategies to improve adherence to LPV. For example, in order to understand how individual clinician characteristics interact with each strategy, we will ask them about their knowledge of LPV and prior experience and comfort with managing MV patients. To understand the role of the inner setting factors, we will query regarding ICU leadership regarding LPV utilization, as well as communication and teamwork between different professional groups involved in MV management. All interviews will be audio-recorded with the participants’ consent and professionally transcribed.

### Data collection methods

As a pragmatic clinical trial, we will capture all patient data electronically via the University of Pennsylvania Health System (UPHS) EHR. Outcomes data will be restricted to data elements that are routinely collected in the course of providing routine clinical care and extracted from the UPHS EHR. We will capture all data regarding contextual elements from interviews and focus groups, as described above.

### Statistical analysis

The primary analytic approach will use a modified intention-to-treat (mITT) approach. Due to EHR constraints with deploying strategies at the ICU level, the clinician nudges included in strategies A and B are only triggered when a new MV order is entered after the time of admission to the ICU. Patients who are initiated on MV before ICU admission (e.g., in the emergency department or due to a clinical emergency on a general ward) may have an MV order entered prior to ICU admission, and therefore may not be exposed to the interventions. Because ICUs differ in their percentages of patients who will and will not be exposed to the strategies, and these patient groups may be systematically different, the primary analysis will exclude patients who have MV ordered prior to the time of admission to an ICU, which we anticipate to be between 10% and 30%, based on preliminary data from study ICUs.

We will also conduct secondary analyses using a sample that will include the true intention-to-treat (ITT) population; that is, all patients who undergo MV in any study ICU (after exclusions detailed above), regardless of whether or not there was exposure to an implementation strategy during the intervention period. This analysis will provide insight into how ICU-targeted strategies may influence the strategies’ effect in implementation outcomes. We hypothesize that the effect of the strategies to improve administration of low tidal volumes will be attenuated in the ITT population.

The unit of analysis for the primary study outcome and all implementation outcomes will be the episode of MV. If a patient has multiple MV episodes, we will include only the first episode during a hospital admission for the primary analysis due to the non-independence of observations within the same patient. The exposure for each patient encounter will be that in place in the admitting ICU at the time of the initiation of MV or ICU admission, whichever is later.

For the primary outcome, we will use mixed effects linear regression models with random effects for ICUs and fixed effects for time to account for the stepped-wedge cluster randomized design. We will make the following 5 comparisons: control vs. strategy A, control vs. strategy B, strategy A vs. strategy B, strategy A vs. strategy A combined with strategy C, and strategy B vs. strategy B combined with strategy C.

Both the mITT and ITT analyses will be conducted with adjustment for the following pre-specified, patient-level covariates that exist prior to randomization: age, gender, height, coronavirus disease 2019 (COVID-19) status, ICU admission source, severity of illness as measured by the Laboratory Acute Physiology Score version 2 (LAPS2) [39], duration of hospitalization prior to ICU admission, duration of ventilation prior to ICU admission, and code status (i.e., the presence or absence of do-not-resuscitate or other treatment-limiting orders). In addition, in recognition of the fact that this trial will begin during the COVID-19 pandemic, we will include a fixed effect for any period of time of alterations in policies affecting mechanically ventilated patients due to COVID-19. Based on prior pandemic policies, this will include any periods of time in a hospital when (1) new, temporary ICUs are opened; (2) any ICUs are closed; and (3) restrictions placed on elective procedure scheduling.

For the effectiveness outcomes of in-hospital mortality and discharge disposition and all adverse outcome measures, we will repeat the analyses as described above, using logistic and linear regression for binary and continuous outcomes, respectively. For the outcomes of duration of MV, ICU LOS, and hospital LOS, death is a competing risk that can further confound the interpretation of results, as interventions that reduce deaths may increase duration of MV and LOS by increased survival of more severely ill patients. We will therefore model these outcomes as composite outcomes of death and MV duration, death and ICU LOS, and death and hospital LOS, ranking the death at the longest/near longest LOS [40]. Because this will produce distributions that will have a spike at the end of the right skewed tail, not mitigated by taking the log of the duration of MV or LOS, we plan to analyze our primary outcome using a simultaneous quantile regression at 20th, 30th, 40th, 50th, 60th and 70th percentiles with random effects. The proposed method will provide effect estimates at each quantile and we will report 95% bootstrap confidence interval (CI). This modelling approach will also allow us to do a joint test of significance across all the quantiles, which is an efficient way of verifying the intervention effect if any.

We will explore patient-level effect modification by repeating the analyses of the primary outcome stratified by two clinical factors that are potential effect modifiers: presence or absence of ARDS and degree of hypoxemia (as measured by the P:F ratio, calculated as the PaO2 divided by the fraction of inspired oxygen [FiO2]). If differences appear in stratified analyses, we will formally evaluate for effect modification by testing the significance of coefficients for multiplicative interaction terms between the potential effect modifier and the study groups on the primary outcome of LPV utilization, and on a subset of effectiveness outcomes: in-hospital mortality, hospital LOS, and days of acute brain dysfunction.

To test the assumptions of our primary analytic approach, we will conduct several sensitivity analyses in which we repeat the primary analysis but with modifications to the study population or how we define the exposure groups or other variables. First, we will include all episodes of MV, including subsequent episodes for the same patient. Second, for patients who are exposed to different study groups within the first 72 h (e.g., due to an inter-ICU transfer or admission during a transition from control period to implementation of a study strategy), we will (1) exclude patients who are exposed to multiple study groups during the first 72 h of MV, and (2) instead define the exposure as the percentage of time during the first 72 h that the patient is exposed to any study groups. Third, a small number of clinicians will staff multiple ICUs, and may become exposed to multiple interventions. We would expect this to result in contamination that would bias the results towards the null. We will estimate the frequency of this occurrence, and will perform a sensitivity analysis excluding patients exposed to clinicians who crossover to multiple ICUs. Fourth, we will include fixed effects (instead of random effects) for ICUs to account for the possibility of ICU-level confounding.

To explore the clinician and environmental contextual factors that may interact with the implementation strategies, two research staff members will code transcripts from interviews. We will use the CFIR framework as a preliminary codebook [35] and add themes and subthemes specific to the data as they arise. We will assess inter-coder reliability by double-coding every fifth transcript. The qualitative analysis will help us to identify the most likely relevant factors associated with implementation of LPV. We will then test these theories using quantitative analyses.

After developing a discrete set of clinician factors from interviews (such as level of experience, assigned to ICU versus rotating responsibilities), we will assign them to clinicians and evaluate whether they modify the effects of the implementation strategies quantitatively using the sample of patients cared for by participating clinicians during the trial. To do so, we will first perform analyses similar to those described above stratified by clinician factors. If we find evidence of differences in the effect sizes, we will add multiplicative interaction terms for each clinician factor with the implementation strategy in the models to quantify whether interactions are significant. To explore the role of ICU factors, we will similarly perform quantitative analyses stratified by ICU. After developing a discrete set of environmental factors from interviews, focus groups, and surveys, we will assign them to ICUs and explore whether any patterns emerge.

All study data will be prepared by the study’s data manager who will extract data from the EHR data warehouse and will be unblinded to study group assignments. She will provide an analytic dataset after deidentification of study ICUs and group assignments to the study’s data analysis (WW). The data analyst and PI will remain blinded to study group assignments during the analysis phase and will responsible for the integrity of the data and the analyses.

### Sample size

We estimated the power of pairwise comparisons of any of the intervention groups (strategy A or B alone or in combination with C—i.e., four separate intervention groups) with the control group. We based our power calculations on the following conservative assumptions: sample size of 8000 episodes across 12 ICUs; an intracluster correlation within ICUs of 0.1; and a baseline mean value of the primary outcome of 45% with a standard deviation of 45% (based on estimates from a retrospective cohort of patients admitted to ICUs in study hospitals during a 6-month period in 2020, including patients with COVID-19). We will use the Holm method to assess the 5 between-arm contrasts of interest by sequentially testing the significance of each against progressively less restrictive alpha levels, preserving the family-wise Type I error rate of 5%. With these assumptions, we estimate that we will have > 95% power to detect an increase of 25% in the mean value of the primary outcome (from 45 to 70%), which would approximate the utilization rates of the study ICU with the highest adherence to tidal volume < 6.0 ml/kg ideal body weight. This sample size also has > 90% power to detect a difference of 20% and nearly 80% power to detect a difference as low as 15% in any pairwise comparisons. Finally, this sample size has 80% power to detect a reduction in in-hospital mortality from an estimated 25 to 16%.

### Study oversight

The INPUT trial was approved by the institutional review boards of the University of Pennsylvania and Chester County Hospital, as detailed further in the “Declarations” section. In compliance with NIH policy, the trial will be overseen by a data safety and monitoring board. Any events of life-threatening acidemia (pH less than 7.10 on the first arterial blood gas after trial enrollment) or of death or cardiac arrest within 24 h of enrollment will be reviewed by a critical care specialist to determine trial relatedness. Any event felt to be possibly or likely trial related will be reported in expedited fashion to the DSMB. In addition, we will perform two interim analyses for review by the DSMB after 9 months and 18 months of two study outcomes: (1) fidelity to LPV (primary outcome, as specified above) and (2) in-hospital mortality. We will not stop the trial early for evidence of effectiveness of the implementation strategies because doing so would reduce our power for secondary analyses and analyses of effect modification. We will propose to stop the trial for early evidence of harm based on in-hospital mortality.

## Discussion

In this paper, we present the protocol for the INPUT study, a pragmatic, stepped-wedge cluster randomized trial of three EHR-based strategies to promote the use of LPV through changing behaviors of clinician stakeholders. To our knowledge, this is one of the first ever large-scale, pragmatic trials embedded within a learning health system that is set up as a hybrid implementation-effectiveness trial with strategies informed by behavioral economics. As a hybrid type III trial, the study will evaluate implementation outcomes primarily and effectiveness outcomes secondarily. We chose this design because of the great strength of evidence for both the efficacy and effectiveness of LPV in several populations of MV patients, and also evidence of a need for improved implementation strategies, making implementation outcomes of great consequence.

This study has several strengths. First, it will rigorously test simple and scalable strategies to improve evidence-based use of a proven-effective, life-saving intervention. As such, it holds great promise to create new knowledge of potentially high impact for patients with a high-risk condition. It will take place in 12 geographically and organizationally distinct ICUs with variable staffing models and patient populations and therefore has high potential for generalizability. Finally, as mentioned, it will provide high-quality evidence to advance the science of implementation in critical care settings through the application of behavioral economics to clinician decision-making [41].

This study also has limitations. First, study hospitals are drawn from a single health system. However, study ICUs employ multiple different staffing models, serve diverse populations of patients in both demographic and clinical terms, and have variability in their baseline rates of adherence to LPV (unpublished data), representative of the diversity of hospitals in the USA, thus supporting the generalizability of the results. Second, because it will be conducted within hospitals with a single integrated EHR, the specific implementation strategies will not be immediately exportable to other health systems with other EHRs. However, because the study health system uses Epic, the most commonly used EHR in the USA, extrapolation of our code (which we will make freely available) to fit other local environments will be facilitated. Third, it is possible that some degree of misclassification could occur in the designation of which patients have ARDS, the condition for which the evidence for LPV is strongest. However, our methods of identifying ARDS have been validated in our health system’s EHR [42], and a primary objective is to generate effectiveness data in a broad population of patients with ARDS, so as to address the challenge of under-recognition of ARDS.

In summary, this stepped wedge trial of strategies to promote LPV among patients who undergo MV will produce generalizable evidence to advance implementation science and behavioral economics to clinician decision-making. If successful, it will furthermore quantify the effectiveness of simple EHR-based strategies with great potential to improve delivery of evidence-based care to patients at high risk for morbidity and mortality.

## Data Availability

Upon completion of the study, any datasets used and/or analyzed during the study will be available from the principal investigator (MPK) in deidentified fashion on reasonable request.

## Abbreviations

APP: Advanced practice provider
ARDS: Acute respiratory distress syndrome
CAM-ICU: Confusion assessment method for the ICU
CFIR: Consolidated framework for implementation research
CI: Confidence interval
COVID-19: Coronavirus disease 2019
CT: Computerized tomography
EHR: Electronic health record
ICU: Intensive care unit
INPUT: Implementation of Nudges to Promote Utilization of low Tidal volume ventilation
IRB: Institutional review board
ITT: Intention-to-treat
LAPS2: Laboratory Acute Physiology Score, version 2
LOS: Length of stay
LPV: Lung-protective ventilation
mITT: Modified intention-to-treat
ml/kg: Milliliters per kilogram
MV: Mechanical ventilation
PBW: Predictive body weight
PEEP: Positive end-expiratory pressure
RASS: Richmond agitation–sedation scale
RT: Respiratory therapist
UPHS: University of Pennsylvania Health System
